# Constructing a prognostic model for hepatocellular carcinoma based on bioinformatics analysis of inflammation-related genes

**DOI:** 10.3389/fmed.2024.1420353

**Published:** 2024-07-11

**Authors:** Yinglian Li, Yuan Fang, DongLi Li, Jiangtao Wu, Zichong Huang, Xueyin Liao, Xuemei Liu, Chunxiao Wei, Zhong Huang

**Affiliations:** ^1^Department of Oncology, Kaiyuan Langdong Hospital of Guangxi Medical University, Nanning, China; ^2^Radiology Department, Guangxi Zhuang Autonomous Region People's Hospital, Nanning, China

**Keywords:** hepatocellular carcinoma, inflammation-related genes, prognostic model, risk score, tumor immune infiltration

## Abstract

**Background:**

This study aims to screen inflammation-related genes closely associated with the prognosis of hepatocellular carcinoma (HCC) to accurately forecast the prognosis of HCC patients.

**Methods:**

Gene expression matrices and clinical information for liver cancer samples were obtained from the Cancer Genome Atlas (TCGA) and the International Cancer Genome Consortium (ICGC). An intersection of differentially expressed genes of HCC and normal and GeneCards yielded inflammation-related genes associated with HCC. Cox regression and the minor absolute shrinkage and selection operator (LASSO) regression analysis to filter genes associated with HCC prognosis. The prognostic value of the model was confirmed by drawing Kaplan–Meier and ROC curves. Select differentially expressed genes between the high-risk and low-risk groups and perform GO and KEGG pathways analyses. CIBERSORT analysis was conducted to assess associations of risk models with immune cells and verified using real-time qPCR.

**Results:**

A total of six hub genes (C3, CTNNB1, CYBC1, DNASE1L3, IRAK1, and SERPINE1) were selected using multivariate Cox regression to construct a prognostic model. The validation evaluation of the prognostic model showed that it has an excellent ability to predict prognosis. A line plot was drawn to indicate the HCC patients’ survival, and the calibration curve revealed satisfactory predictability. Among the six hub genes, C3 and DNASE1L3 are relatively low expressed in HCCLM3 and 97H liver cancer cell lines, while CTNNB1, CYBC1, IRAK1, and SERPINE1 are relatively overexpressed in liver cancer cell lines.

**Conclusion:**

One new inflammatory factor-associated prognostic model was constructed in this study. The risk score can be an independent predictor for judging the prognosis of HCC patients’ survival.

## 1 Introduction

Worldwide, hepatocellular carcinoma (HCC) is the most common malignant tumor. HCC patients are mostly at an advanced stage when diagnosed and have a poor prognosis (1). The most severe result of uncontrolled hepatocyte growth is the occurrence of HCC, whose progression and metastasis are inseparable from the deterioration of the liver regeneration microenvironment (2). The drug therapy for HCC encompasses a variety of treatment modalities. As the first FDA-approved systemic therapy for advanced HCC, Sorafenib remains a cornerstone treatment, primarily due to its ability to extend survival (3, 4). Following Sorafenib, other drugs such as Regorafenib, Cabozantinib, and Lenvatinib have been approved for use (5). These drugs target various tumor growth pathways and angiogenesis pathways (3, 5). Drugs like Nivolumab and Pembrolizumab have shown promise in treating HCC by enhancing the body’s immune response against tumor cells (6). Combining Sorafenib with immune checkpoint inhibitors may improve treatment efficacy (7, 8). However, due to the insidious nature of HCC development, high recurrence rate after surgical resection, and high transplantation failure rate, improving the prognosis of HCC patients and identifying new molecular targets for drug development still faces multiple challenges, including those involved in cancer metabolism, immune evasion, and cell survival (9).

Chronic inflammation caused by viral infection significantly increases the possibility of HCC development by activating inflammatory signaling pathways and cytokines (10). The inflammatory pathways are complex and highly interconnected with multiple feedback loops and interactions. This complexity makes it difficult to predict how modulating one pathway might affect others, potentially leading to unintended consequences (11, 12). Thus, inflammation is a significant driver of cancer progression. The expression and role of inflammation-related genes can differ significantly among tumors, complicating the development of universally effective treatments (13, 14). Tumor cells can develop resistance to therapies that target inflammation-related pathways, often through genetic mutations or by activating alternative pathways (15). This adaptation can reduce the long-term efficacy of these treatments (16). Developing computational models that simulate individual responses to therapies targeting inflammation-related genes may help optimize treatment strategies (17, 18). Integrating inflammation-related genes into tumor therapy for conditions like HCC presents significant opportunities. Exploring the correlation between inflammation-related genes and tumor immune status may help further integrate targeted therapy and immunotherapy (19, 20).

Immune inflammation involves the immune system’s response to cancer cells, which can either suppress tumor growth or contribute to tumor development and progression (21). Inflammation-related genes are crucial in this context as they can influence the tumor microenvironment, affecting the behavior of HCC. HCC is a highly heterogeneous disease, and the genetic profile can vary significantly among patients, complicating creating a universally applicable model (22). Some studies have identified and evaluated the potential prognostic value of immune-autophagy-related genes in HCC patients (23). The study’s assessment was based on the complex interplay between the immune system, autophagy processes, and the tumor microenvironment in liver cancer (24). However, further research is still needed to explore the complex interactions between inflammation-related genes and other pathways in HCC. We identify inflammation-related genes that are significantly altered in HCC. Integrate data from different sources and use statistical and machine learning models to analyze the relationship between the expression of inflammation-related genes and patient survival. Techniques such as the proportional hazards model (25) can be used to estimate risk based on gene expression levels. Due to a more aggressive tumor microenvironment, certain patients with high expression of pro-inflammatory genes may have a poorer prognosis. We also used models to classify patients into risk groups and examine their immune microenvironment. Our model can predict patient survival while helping clinicians plan treatment more efficiently.

## 2 Materials and methods

### 2.1 Data acquisition and preparation

This study downloaded the normalized RNA-Seq data set of 371 HCC samples and 50 adjacent normal samples and the corresponding clinical information from the UCSC Xena browser.^1^ The clinical information includes follow-up time, survival status, age, gender, TNM stage, and overall stage of HCC patients. Download the LIRI-JP HCC data set of the ICGC database from the Sangerbox platform^2^ as an independent validation set for the prognostic model containing RNA-Seq data of 240 HCC tumor samples and corresponding clinical information. The sample data uses standardized count values. Inclusion and exclusion processing criteria: (1) exclude samples without clinical follow-up data; (2) exclude those without TTL data; (3) exclude those without information related to patient survival status; (4) convert ENSEMBL ID to gene symbols, and (5) If multiple gene symbolic expressions exist, the median value is recorded. The clinical data of these samples are shown in Supplementary Table S1. Inflammation-related genes were searched in the GeneCard database using the keyword “inflammation.”

### 2.2 Screening of differentially expressed inflammation-related genes related to HCC

RStudio version 4.1.0 and the “Limma” software package (26) were used to screen differentially expressed genes (DEGs) between HCC and adjacent normal samples. First, genes with an average count of less than one were excluded, and genes were screened based on the criteria that the absolute value of the Log2-transformed fold change (FC) was more significant than or equal to 4, and the significance *p* value was less than 0.01. The genes that met the conditions were selected as DEGs, among which log2FC greater than 4 is an up-regulated gene, and less than −4 is a down-regulated gene. Use the “ggplot2” R software package (27, 28) to draw a volcano plot to visualize the results. Next, a correlation coefficient greater than six was used as the screening criterion to obtain inflammation-related genes from the GeneCards database (29). Finally, the intersection of DEGs and inflammation-related genes was used to obtain differentially expressed inflammation-related genes in HCC, and Venny online software^3^ (30) was used to draw a Venn diagram for visualization. The “survival” R software package (31) performed Univariate Cox regression analysis (25) on differentially expressed inflammation-related genes in HCC. Genes with *p* less than 0.05 were differentially expressed inflammation-related genes related to prognosis. This gene is a risk factor for the prognosis of HCC (The hazard Ratio (HR) > 1). This gene is a protective factor for the prognosis of HCC(HR < 1).

### 2.3 Least absolute shrinkage and selection operator regression and construction of the prognostic model

The 50 paracancerous and HCC samples with missing survival information downloaded from the TCGA database were eliminated, and 365 hepatocellular carcinoma samples were retained as a training set for constructing the prognostic model. To further screen variables, the “glmnet” R software package (32) was used to perform Lasso (Least absolute shrinkage and selection operator is a type of linear regression that includes a penalty equal to the absolute value of the magnitude of coefficients.) regression analysis on the differentially expressed inflammation-related genes related to HCC prognosis screened out in the above Univariate Cox regression analysis to reduce the purpose of the fitting degree of the constructed prognostic model. During the Lasso regression analysis process, 10-fold cross-validation was used to determine the λ value, and the λ with the minor partial likelihood deviation was selected as the optimal λ. Cross-validation in Lasso regression ensures the model is tuned for optimal performance by finding the best regularization parameter (alpha). K-Fold Cross-Validation: Choose the number of folds (typically 5 or 10). Split the dataset into 𝑘 folds. Each fold will be used once as a validation set, while the remaining 𝑘−1 folds form the training set. The genes screened by Lasso regression analysis in the previous step were further used to perform Multivariate Cox regression analysis using the “survival” R software package. In the Multivariate Cox regression analysis results, genes with *p* less than 0.05 were considered independent factors affecting the prognosis of HCC patients. The coefficients for Multivariate Curve Resolution (MCR) analysis (33) were adopted for calculating the RS.

In the TCGA-LIHC cohort, the samples were assigned to a high-risk or low-risk group (cut-off: 50%). The risk score of each HCC patient was calculated according to the prognostic model, and the patients were divided according to the median risk score. Patients whose risk score was higher than the median risk score were divided into high-risk groups. Next, the “survival” software package is used to draw the Kaplan–Meier curve(a statistical tool used in survival analysis to estimate the survival function from lifetime data); the “survival,” “survminer,” and “timeROC” software packages (22) are used to evaluate the prognosis. The ability of the model to predict the 1-year, 2-year, and 3-year survival rates of HCC patients. Finally, the ROC curves of the 1-year, 2-year, and 3-year survival rates of HCC patients in the prediction training set of age, gender, TNM stage, total stage, and risk score prediction training set were plotted to compare the corresponding AUC values further to illustrate the predictive ability of the prognostic model. Clinicopathological characteristics, including age, gender, TNM stage, total stage, and risk score, were integrated, and the nomogram was constructed using “rms” packages in R.

Akaike information criterion (AIC) (34) is a standard for evaluating the complexity of a statistical model and measuring the goodness of fit of a statistical model to the data. AIC can be expressed as AIC = 2 k − 2ln(L). Where k is the number of parameters and L is the likelihood function. When the complexity of the model increases (k increases), the likelihood function L will also increase, thereby making the AIC smaller. However, when k is too large, the growth rate of the likelihood function slows down, causing the AIC to increase. If the model is too complex, it is easy to cause an Overfitting phenomenon.

### 2.4 Gene enrichment analysis

To better understand the biological functions of DEGs between high-risk groups and low-risk groups in the training set, four R software packages: “clusterProfiler” (35), “org.Hs.eg.db” (36), “enrich plot,” and “GOplot” (37) were used. For GO and KEGG functional enrichment analysis, *p* < 0.05 after correction was set as the screening condition.

### 2.5 Tumor-infiltrating immune cells

To evaluate the difference in immune infiltration of tumor tissue between high-risk and low-risk groups in the training set and the association between risk score and immune infiltration of tumor tissue. This study used a gene expression matrix to perform ESTIMATE (38) analysis to calculate each sample’s stromal score, immune score, ESTIMATE score, and tumor purity. The TIMER algorithm calculated each sample’s infiltration of six tumor-infiltrating immune cell subsets (B cells, CD4+ T cells, CD8+ T cells, macrophages, neutrophils, and dendritic cells). Finally, the correlation between the risk score calculated based on the prognostic model and tumor immune infiltration was analyzed.

### 2.6 Cell line

The normal human liver cell line LO2 and two human hepatocellular carcinoma cell lines (97H and HCCLM3) were purchased from Saiku Biotech Co. The complete medium for culturing cells was prepared using Dulbecco’s modified medium (DMEM) with 10% fetal calf serum and 1% double antibody (penicillin/streptomycin).

### 2.7 Real-time fluorescence quantitative PCR experimental materials

The human regular liver cell line is LO2, and there are two human liver cancer cell lines (97H and HCCLM3). The complete medium for culturing cells was prepared using Dulbecco’s modified medium (DMEM) with 10% fetal calf serum and 1% double antibody (penicillin/streptomycin). Total cellular RNA was extracted using the TRIZOL method. The concentration of extracted RNA was measured by NanoDrop2000 (UV spectroscopy) and was not less than 50ug. Intact total RNA produces clear 28S and 18S rRNA bands when subjected to denaturing gel electrophoresis (2:1). Carry out the entire reverse transcription reaction according to the instructions of TaKaRa Reverse Transcription Kit PrimeScrip™ RT reagent Kit with gDNA Eraser (Perfect Real Time), shown in Supplementary Table S2. Dissolve the FastStart Universal SYBR Green Master (ROX), the upstream primer (Forward primer), and the downstream primer (Reverse primer) on ice for later use. Follow the instructions to prepare the components and configure a 10 μL reaction system. Configure a main tube according to the above features, mix gently with a pipette and centrifuge, and add samples sequentially. Set 4 duplicate wells for each sample. The real-time fluorescence quantitative PCR (qTOWER^3^ Series, Analytik Jena, Germany) reaction procedure, shown in Supplementary Table S3, is described in the instructions. The DNA solubilization curve verifies the specificity of the amplification product. From 60 to 98 degrees, the horizontal coordinates of the curve are each temperature point. The vertical coordinate is the change in fluorescence intensity. When the amplification rate of the target genes (C3, CTNNB1, CYBC1, DNASE1L3, IRAK1, SERPINE1) reaches 100%, the cycle threshold is obtained [Linear dynamic range (12–30)] and the relative expression of the target gene mRNA is calculated using the 2^−△△Ct^ method. Experimental results were visualized using GraphPad Prism 8 software. The specific primer sequences are shown in Table 1. The suppression test detects the extraction situation. 3 samples using the same method of repeatability (Intra-Assay Variation) to assess the intra-assay variation.

**Table 1:** Specific primer sequence.

Gene	Upstream primer	Downstream primer
GAPDH	TGACTTCAACAGCGACACCCA	CACCCTGTTGCTGTAGCCAAA
DNASE1L3	TGGTTGAGGTCTACACGGACGT	GTCAGTCCTCAAGCGGATGTTC
C3	TCACCGTCAACCACAAAGCTGCTACC	TTTCATAGTAGGCTCGGATCTTCCA
CTNNB1	GGCTCTTGTGCGTACTGTCCTTC	CTTGGTGTCGGCTGGTCAGATG
CYBC1	TGTGAGCGTGGAGGAGGAGAAG	CTGGTGATGAGCTTGGCGATGG
IRAK1	GACACGGACACCTTCAGCTTTGG	CAGCCTCCTCAGCCTCCTCTTC
SERPINE1	GGTGCTGGTGAATGCCCTCTAC	TGCTGCCGTCTGATTTGTGGAAG

### 2.8 Statistical method

All statistical analyses were performed using R software (v4.1.0). The stepwise regression method was used to screen Hub genes to construct a prognostic model. The Kaplan–Meier curve compared the prognostic differences between HCC patients in the high-risk and low-risk groups. The *t*-test was used to compare the differences in risk scores between different clinicopathological characteristic groups. *p* < 0.05 (two-sided) was considered statistically significant.

## 3 Results

### 3.1 Screening of differentially expressed inflammation-related genes in HCC

**Figure 1:**
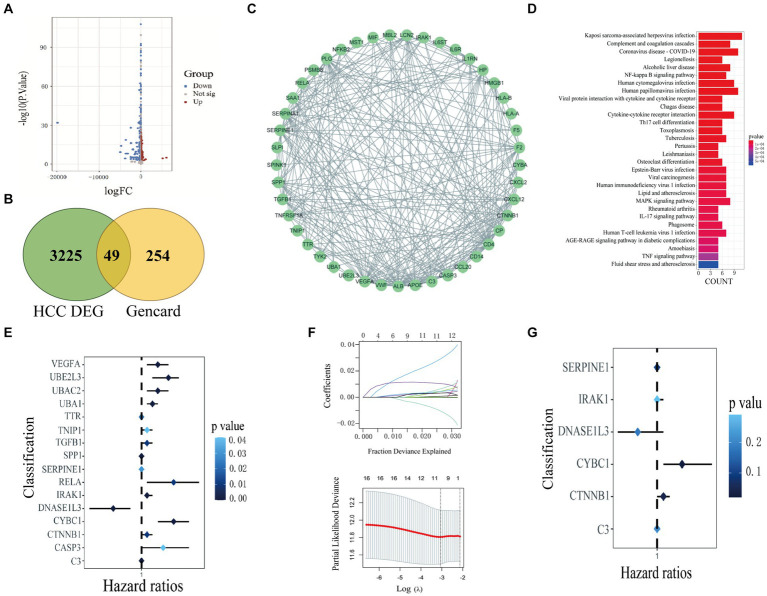
Screening of inflammation-related genes in HCC. **(A)** Volcano plot of DEGs in liver cancer; **(B)** Venn diagram of DEGs and inflammation-related genes; **(C)** PPI network of inflammation-related genes in liver cancer. **(D)** KEGG analysis of inflammation-related genes. **(E)** The risk ratio forest plot showed the prognostic value of the genes based on univariate Cox regression analysis. **(F)** Lasso regression analysis and Cross-validation. **(G)** The risk ratio forest plot showed the prognostic value of the genes based on multivariate Cox regression analysis.

Through differential expression analysis, 3,274 DEGs between HCC samples and normal liver samples were obtained, including 2,789 up-regulated genes and 485 down-regulated genes, as shown in the volcano plot in Figure 1A. 303 inflammation-related genes were retrieved from the GeneCards database. Then, we take the intersection between the database and differential genes and call these 49 intersection genes inflammation-related genes (Figure 1B). Then, we used the STRING database (39) to construct a Protein–Protein Interaction (PPI) network of HCC inflammation-related genes. The interaction score was set to 0.7 during PPI analysis, and Cystoscope software visualized the results (Figure 1C).

### 3.2 GO and KEGG enrichment analysis

We performed GO and KEGG functional enrichment analysis on 49 inflammation-related genes. GO analysis results show that cell functions are significantly enriched in positive regulation of cytokine production, secretory granule lumen, cytokine receptor binding, etc. (Supplementary Figure S1); KEGG pathway analysis shows that it is mainly enriched in NF-κB signaling pathway, cytokine-cytokine receptor interaction, Th17 cell differentiation, etc. (Figure 1D).

### 3.3 Screening of prognostic model hub genes and construction of a prognostic model

**Figure 2:**
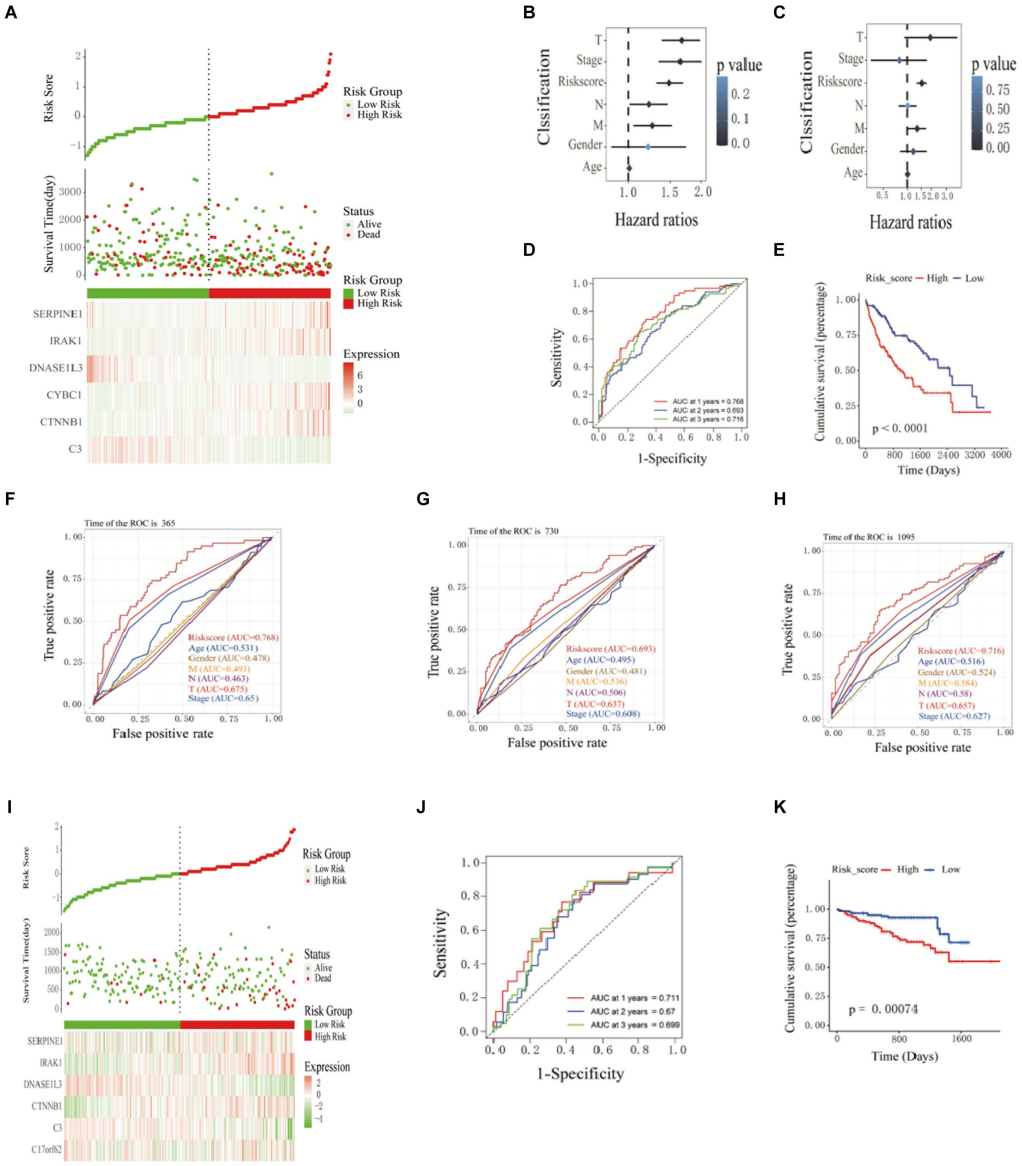
Construction and validation of prognostic models. **(A)** Prognostic distribution of HCC, differences in survival status between high-risk and low-risk groups, and heat maps of the expression profiles of the six hub genes. The risk score and clinicopathological features are subjected to univariate **(B)** and multivariate **(C)** Cox regression analysis, as illustrated in the forest plot. **(D)** Kaplan–Meier survival curves and **(E)** time-dependent ROC curves. ROC curve verified the significance of the prognostic model **(F)** 365 days, **(G)** 730 days, and **(H)** 1,065 days. **(I)** Distribution of prognostic index. Survival status of patients in different groups. Heat map of the expression profile of the included inflammation-related genes. **(J-K)** Kaplan-Meier survival curves and time-dependent ROC curves.

Based on the prognostic information of tumor samples in the training set, univariate Cox regression analysis (*p* < 0.05) was performed on 49 differentially expressed inflammation-related genes in HCC, and 16 genes significantly related to the prognosis of HCC were screened out (C3, CASP3, CTNNB1, CYBC1, and DNASE1L3), (IRAK1, RELA, SERPINE1, SPP1, TGFB1, TNIP1, TTR, UBA1, UBAC2, UBE2L3, and VEGFA), as shown in Figure 1E. Then, Lasso regression analysis was further performed on 16 inflammation-related genes related to the prognosis of HCC patients to obtain 11 genes (C3, CTNNB1, CYBC1, DNASE1L3, IRAK1, SERPINE1, SPP1, UBA1, UBAC2, UBE2L3, and VEGFA), as shown in Figure 1F. Finally, the above 11 genes were screened using the Step calculation function in multifactor Cox regression analysis based on the AIC information statistic. When AIC was equal to 1316.19, the results were obtained based on 6 Hub genes (C3, CTNNB1, CYBC1, DNASE1L3, the optimal prognostic model constructed by IRAK1, SERPINE1). As shown in Figure 1G, multivariate Cox regression risk showed that CYBC1 (HR = 1.04, 95%CI = 1.01–1.09, *p* = 0.02) among the six Hub genes was an independent prognostic risk factor for HCC patients. A prognostic model is constructed through regression coefficients. In the prognostic model, the risk score calculation formula for each HCC sample is Risk score = (−0.000337128 × C3) + (0.009464722 × CTNNB1) + (0.045920338 × CYBC1) + (−0.035384453 × DNASE1L3) + (0.007516301 × IRAK1) + (0.001958146 × SERPINE1). The risk scores of all HCC patients in the training set were calculated according to the prognostic model formula. Patients with risk scores higher than the median were classified into the high-risk group and others as the low-risk group. Figure 2A shows that the risk scores of HCC patients gradually increase from left to right. It can be seen from Figure 2A that as the risk score increases, the survival time of patients in the high-risk group is shorter (the trend of concentration of scattered points is downward), and the mortality rate is higher (red dots increase). Figure 2A shows the differential expression of six Hub genes between the high-risk and low-risk groups in the form of a heat map. The expression of four genes, SERPINE1, IRAK1, CTNNB1, and CYBC1, was relatively up-regulated in the high-risk group. The expression of C3 and DNASE1L3 Expression was moderately upregulated in the low-risk group.

### 3.4 Verification of pivot genes

The patients’ risk scores in the training set were comprehensively analyzed with the corresponding clinicopathological characteristics (age, gender, TNM stage, and total stage). Single-factor Cox regression analysis showed that the risk score (*p* < 0.001), M stage (*p* = 0.01), N Stage (*p* = 0.04), T stage (*p* < 0.001), and total stage (*p* < 0.001) are significantly related to the prognosis of HCC (Figure 2B). Multivariate Cox regression analysis showed that risk score (HR = 1.52, 95%CI = 1.31–1.75, *p* < 0.001) and M stage (HR = 1.33, 95%CI = 1.02–1.72, *p* = 0.03) are essential factors affecting the prognosis of HCC patients. Independent risk factors (Figure 2C). The ROC curve was used to evaluate the accuracy of the prognostic model, and the AUC values for predicting the 1-, 2-, and 3-year survival rates of HCC patients in the training set were 0.768, 0.693 and 0.716, respectively (Figure 2D). The Kaplan–Meier curve showed that the prognosis of the high-risk group was worse than that of the lower-risk group, and the difference was statistically significant (*p* < 0.0001) (Figure 2E). The ROC curve(is a graphical plot used to evaluate the performance of a binary classifier system. It plots the true positive rate (TPR) against the false positive rate (FPR) at various threshold settings) and was used to verify the predictive performance of the prognostic model. In the ROC curve predicting the 1-year (Figure 2F), 2-year (Figure 2G), and 3-year (Figure 2H) survival rates of HCC patients in the training set, the AUC value of the risk score was always high. Compared with clinicopathological characteristics such as age, gender, TNM stage, and total stage, it indicates that the risk score may be a reliable predictor of the prognosis of HCC patients. 240 HCC samples from the ICGC database were used as a validation set further to verify the reliability and accuracy of the model. The same formula was used to calculate the risk scores of the 240 HCC samples, and patients higher than the median risk score were divided into high-risk groups; otherwise, they were split into low-risk groups. The risk score distribution, survival time, and mRNA expression level distribution of the six Hub genes of the patients in the validation set are shown in Figure 2I. The AUC values for predicting 1-year, 2-year, and 3-year survival rates were 0.711, 0.67, and 0.699, respectively, (Figure 2J). The Kaplan–Meier curve showed that the prognosis of patients in the high-risk group was worse than that of the lower-risk group, and the difference was statistically significant (*p* = 7.4e-4) (Figure 2K). The research results of the validation and training sets are consistent, indicating that this prognostic model can effectively predict the prognosis of HCC patients.

### 3.5 Immune infiltration analysis of high and low-risk groups

**Figure 3:**
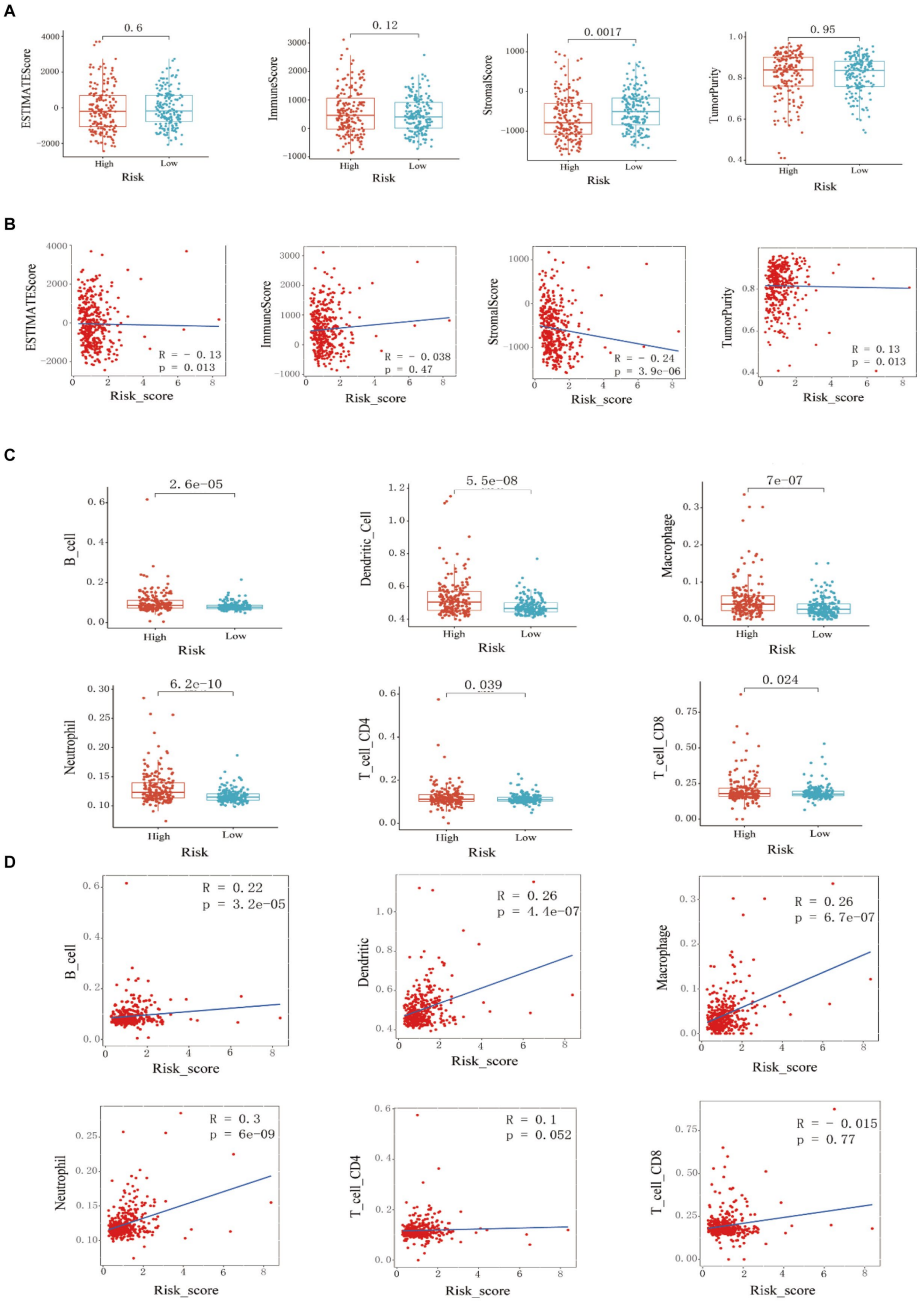
Training set immune infiltration analysis. **(A)** Analysis of differences in ESTIMATE scores, immune cell scores, stromal cell scores, and **(B)** tumor purity between high-risk and low-risk groups, and correlation analysis with risk scores. **(C)** Analysis of differences in the infiltration abundance of B cells, macrophages, neutrophils, dendritic cells, CD4+ T cells, and CD8+ T cells between high-risk and low-risk groups, and **(D)** correlation analysis with their respective risk scores.

ESTIMATE analysis found that the stromal cell score difference between the high and low-risk groups in the training set was statistically significant, and the low-risk group had a higher stromal cell score (*p* < 0.001). There was no statistically significant difference in immune cell score, ESTIMATE score, and tumor purity between the high-risk and low-risk groups (Figure 3A). By studying the correlation between the risk score model and the four scores, it was found that the stromal cell score was negatively correlated with the risk score (R = −0.24, *p* = 3.9e-06); the ESTIMATE score was negatively correlated with the risk score (R = −0.13, *p* = 0.013); tumor purity was positively correlated with risk score (R = 0.13, *p* = 0.013) (Figure 3B). Through TIMER analysis, the infiltration abundance of B cells, macrophages, neutrophils, dendritic cells, CD4+ T cells, and CD8+ T cells in HCC was higher in the high-risk group than in the lower-risk group (*p* < 0.05) (Figure 3C). By analyzing the correlation between risk score and immune cell infiltration abundance, it was found that B lymphocytes were positively correlated with risk score (R = 0.22, *p* = 3.2e-05); dendritic cells were positively correlated with risk score (R = 0.26, *p* = 4.4e-07); macrophages were positively correlated with risk score (R = 0.26, *p* = 6.7e-07); neutrophils were positively correlated with risk score (R = 0.3, *p* = 6e-09) (Figure 3D).

### 3.6 Combining clinical data to construct an HCC prognostic nomogram

**Figure 4:**
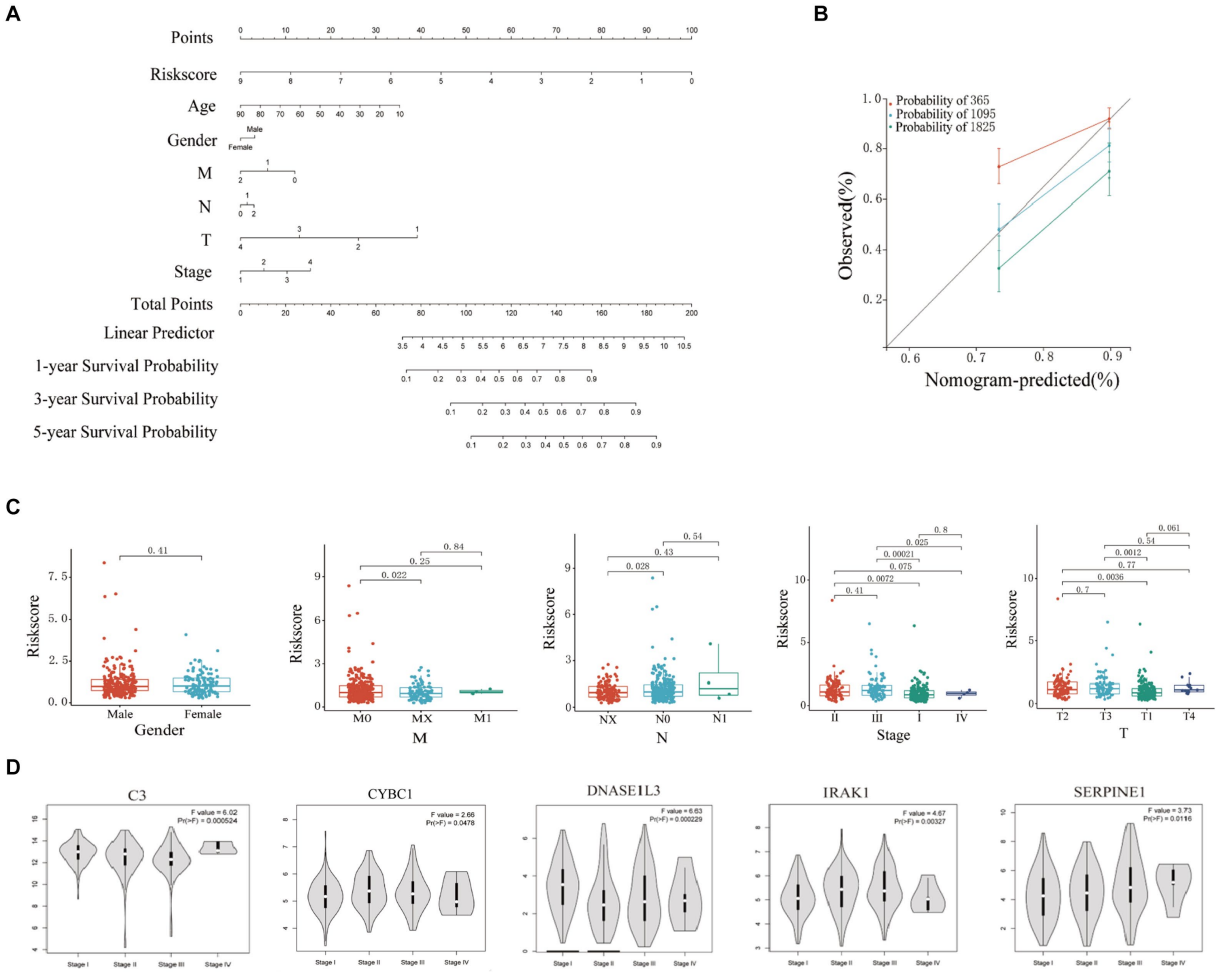
Clinical correlation analysis based on training set risk score. **(A)** Nomogram model to predict 1-, 3-, and 5-year survival rates of HCC cases. **(B)** Calibration graphs indicated that predicted 1-, 3-, and 5-year survival rates were close to the actual survival rates. **(C)** Results of correlation analysis between risk score and gender, TNM, and stage. **(D)** Analyze the correlation between the mRNA expression of 6 hub genes based on the GEPIA.

In this study, the nomogram constructed by combining the clinicopathological characteristics and risk scores of HCC patients in the training set was used to predict the 1-year, 3-year, and 5-year survival probabilities of HCC patients (Figure 4A). C-index equals 0.697 (greater than 0.5). The 3-year actual survival rate was highly consistent with the predicted value, indicating that the constructed nomogram had good accuracy (Figure 4B). In this study, the risk score was closely related to the TNM stage and total Stage, indicating that the prognostic model helps predict the proliferation and metastasis of HCC (Figure 4C). The correlation between the expression of six Hub genes (C3, CYBC1, CTNNB1, DNASE1L3, IRAK1, and SERPINE1) constructed for prognostic modeling and the level of immune cell infiltration was analyzed using the TIMER database. The results showed that the expression of C3 in HCC was closely correlated with the infiltration of B cells, CD4+ T cells, macrophages, neutrophils, and dendritic cells (*p* < 0.05). The expression of CTNNB1 in HCC was closely correlated with the infiltration of B cells, CD4+ T cells, CD8+ T cells, and macrophages (*p* < 0.05). The expression of CYBC1 in HCC was closely correlated with the infiltration of B cells, CD4+ T cells, macrophages, neutrophils, and dendritic cells (*p* < 0.05). DNASE1L3 expression in HCC was closely associated with infiltration of B cells, CD4+ T cells, CD8+ T cells, neutrophils, and dendritic cells (*p* < 0.05). IRAK1 expression in HCC was closely associated with infiltration of B cells, CD8+ T cells, macrophages, and dendritic cells (*p* < 0.05). The expression of SERPINE1 in HCC was closely associated with the infiltration of CD8+ T cells, macrophages, neutrophils, and dendritic cells (*p* < 0.05). These results suggest that the prognostic model Hub genes are closely associated with immune infiltration, and the results are shown in Supplementary Figure S2, with *p* < 0.05 indicated by red boxes. The GEPIA database was further analyzed for six Hub genes, mRNA expression of CYBC1 (HR = 1.7, Logrank *p* = 0.0036), DNASE1L3 (HR = 0.43, Logrank *p* = 2e-06), IRAK1 (HR = 1.7, Logrank *p* = 0.0042), SERPINE1 (HR =1.5, Logrank *p* = 0.027) mRNA expression correlated with overall survival of hepatocellular carcinoma patients as shown in Supplementary Figure S3. The mRNA expression of C3 (*F* = 6.02, *p* = 0.000524), CYBC1 (*F* = 2.66, *p* = 0.0478), DNASE1L3 (*F* = 6.63, *p* = 0.000229), IRAK1 (*F* = 4.67, *p* = 0.00327), SERPINE1 (*F* = 3.73, *p* = 3.73) and SERPINE1 (F = 3.73, *p* = 0.0116) was statistically significant among the groups with different hepatocellular carcinoma clinical staging as shown in Figure 4D.

### 3.7 Expression in human hepatocellular carcinoma cells and normal cells of the liver

**Figure 5:**
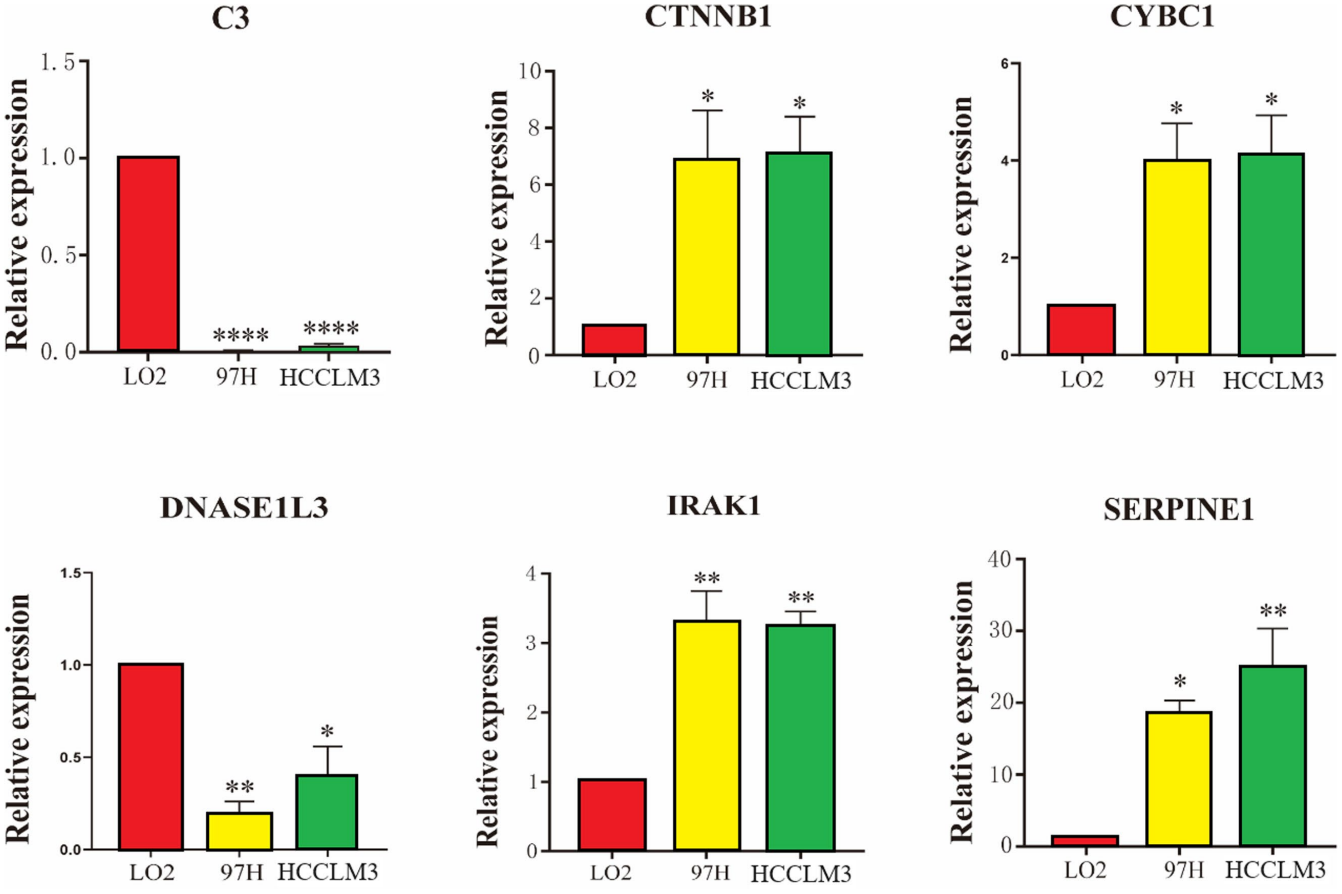
Comparison of the mRNA expression levels of six modeled genes in hepatocellular carcinoma and normal liver cells.

As shown in Figure 5, the expression differences of the six Hub genes C3, CTNNB1, CYBC1, DNASE1L3, IRAK1, and SERPINE1 between human liver cancer cell lines and normal liver cell lines are statistically significant. C3 and DNASE1L3 are significantly different in HCCLM3 and 97H liver cancer cells. The cell lines showed relatively low expression compared with normal liver cells, while CTNNB1, CYBC1, IRAK1, and SERPINE1 showed relatively high expression in HCCLM3 and 97H liver cancer cell lines compared with normal liver cells.

## 4 Discussion

Studies are developing and validating a systemic immune-inflammatory index based on lymphocyte, neutrophil, and platelet counts and exploring its prognostic value in HCC (40). Other studies have found the prognostic value of prognostic nutritional index (PNI) and systemic immune-inflammatory index in hepatocellular carcinoma (41). Studies have reviewed the utility of inflammatory markers as prognostic tools in patients with resectable HCC (42). It is believed that there is currently a lack of reliable prognostic biomarkers to predict postoperative recurrence of HCC. However, we showed a prognostic model based on six inflammation-related genes, including C3, CTNNB1, CYBC1, DNASE1L3, IRAK1, and SERPINE1, which was established through multi-factor Cox regression analysis. According to the formula, the risk score of each patient is calculated and used as a standard to predict the outcome of HCC patients. The study found that High-risk scores among HCC patients have a worse prognosis than those in the low-risk group. ROC curve analysis of the survival rate of HCC patients found that the risk score has good sensitivity and specificity. The AUC values for predicting 1-year, 2-year, and 3-year survival rates are 0.768, 0.693, and 0.716, respectively, which can be used to predict prognosis for HCC patients. In addition, the predictive ability of the constructed prognostic model was successfully verified using the HCC data set of the ICGC database.

Current studies suggest that changes in the C3 gene and its expression can affect tumor immune response. Excessive activation or inhibition of complement components may lead to tumor progression or immune escape. At the same time, chronic inflammation caused by chronic hepatitis B is usually mediated by complement activation and is a known risk factor for liver cancer. Elevated levels of C3 and other complement components have been observed in HCC patients. Changes in the levels of complement proteins (including C3) in the blood of HCC patients may serve as biomarkers for the diagnosis or prognosis of HCC (43–46). The CTNNB1 gene encodes β-catenin and is involved in the regulation and coordination of cell–cell adhesion and gene transcription. Mutations in the CTNNB1 gene in HCC often lead to the stabilization and accumulation of β-catenin in the nucleus and abnormal activation of the Wnt/β-catenin signaling pathway is associated with the pathogenesis of HCC. HCC tumors with CTNNB1 mutations often show unique histological features and may have a better prognosis than HCC without these mutations. The level and activity of β-catenin represented by CTNNB1 can serve as a biomarker for diagnosing and classifying HCC. The presence of CTNNB1 mutations can also affect the prognosis of HCC patients. Studies have shown that patients with CTNNB1 mutant HCC may respond better to certain treatments, and the role of CTNNB1 in HCC helps develop targeted therapies aimed at inhibiting the Wnt/β-catenin signaling pathway. At the same time, targeting β-catenin signaling with other treatments (such as immune checkpoint inhibitors or traditional chemotherapy) can provide a more effective treatment strategy for HCC patients (47–53). The CYBC1 gene encodes a protein that acts as a chaperone for cytochrome b-245, which is a component of the NADPH oxidase complex. The CYBC1-cytochrome b-245 complex is essential for producing ROS, and NADPH oxidase and its ROS can affect cancer’s development and progression. In chronic inflammation caused by HCC, dysregulated ROS production, which CYBC1 may affect, may lead to a pro-inflammatory environment and promote the development of liver cancer. Alterations in CYBC1 expression or function may affect the immune system’s ability to respond to tumor cells (54, 55). The DNASE1L3 gene encodes an enzyme that digests extracellular DNA released during cell death processes such as apoptosis and necrosis. Clearing these DNA fragments maintains homeostasis and prevents excessive inflammation. Chronic inflammation is an important risk factor for liver cancer. The normal function of DNASE1L3 may help maintain genomic stability by preventing the accumulation of DNA fragments, which may otherwise lead to mutations and cancer. Changes in DNASE1L3 expression or activity may serve as a biomarker for HCC (56–62). IRAK1 is a serine/threonine protein kinase that participates in downstream signaling of the IL-1 and TLR pathways. It is essential for activating nuclear factor kappa B (NF-κB) and mitogen-activated protein kinase (MAPK) signaling. Through its role in IL-1 and TLR signaling, IRAK1 can promote the inflammatory microenvironment that promotes the development of liver cancer. Abnormal activation of IRAK1 can lead to sustained activation of the NF-κB and MAPK pathways, promoting cell proliferation, survival, and anti-apoptosis. Elevated IRAK1 expression or activity levels may serve as a biomarker for HCC. At the same time, studies have shown that high IRAK1 expression is associated with poor prognosis in HCC patients (63–66). SERPINE1 (serine protease inhibitor family E member 1) is a gene that encodes a protein that regulates fibrinolysis and plays a role in cell migration, invasion, and angiogenesis. Elevated levels of SERPINE1 are associated with increased tumor growth, invasion, and metastasis in multiple cancers, including HCC. It also plays a role in angiogenesis (forming new blood vessels), essential for tumor growth and metastasis. It interacts with vitronectin and integrins, influencing endothelial cell migration and blood vessel formation. High expression of SERPINE1 is often associated with poor prognosis in HCC patients (67–72). In summary, the six Hub genes in the prognostic model have been confirmed in multiple studies to be involved in the occurrence and development of HCC.

This study also showed a close correlation between HCC and stromal cells (73, 74). ESTIMATE (75) analysis showed that the stromal score in the low-risk group was significantly higher than in the high-risk group, and the difference was statistically significant. Correlation analysis showed that stromal cell and ESTIMATE scores negatively correlated with risk scores. Dense immune cell infiltration is generally associated with a better prognosis because it indicates that the immune system is actively responding to the presence of the tumor. Lower immune cell infiltration or an immune desert state (few immune cells in the tumor microenvironment) is often associated with a poorer prognosis because it indicates that the tumor may have evaded surveillance by the immune system (76). TIMER’s (77) algorithm analysis found that the infiltration of B lymphocytes, macrophages, dendritic cells, and neutrophils in HCC tumor cells was positively correlated with the risk score. Immune cell infiltration plays an important prognostic role in hepatocellular carcinoma (HCC). Studies have shown that immune cells’ composition and infiltration level in the tumor microenvironment are closely related to the patient’s prognosis (78). Researchers have developed prognostic models based on immune cell infiltration by integrating large-scale and single-cell RNA sequencing data. These models can predict the survival rate and immune checkpoint blockade response of HCC patients (79). The distribution and activity status of different types of immune cells, such as T cells, B cells, and macrophages in the tumor microenvironment, are key prognostic indicators. High levels of anti-tumor immune cells (such as cytotoxic T cells) are generally associated with a better prognosis. In contrast, immunosuppressive cells (such as regulatory T cells) may indicate a poor prognosis (80). Multiple studies have evaluated the effect of immune cell infiltration on HCC prognosis through survival analysis and found that patients with higher immune scores generally have longer survival (52, 78, 81, 82). These patients tend to have higher anti-tumor immune cell infiltration and lower immunosuppressive cell infiltration (83). Studies have shown that combining ICIs with other treatments, such as chemotherapy, radiotherapy, or targeted therapy, can enhance the therapeutic effect. For example, the effect of immunotherapy can be enhanced by inhibiting immunosuppressive cells or promoting the infiltration of anti-tumor immune cells. Suppose there are more regulatory T cells (Tregs) and M2 macrophages in the tumor microenvironment (84, 85). In that case, this may promote tumors to escape immune surveillance, thereby increasing the risk of tumor survival and growth. The high density of cytotoxic T cells (such as CD8+ T cells) and M1 macrophages may enhance the ability to attack tumors and promote the clearance of tumor cells, thereby potentially reducing tumor invasiveness and patient risk (86). Immunotherapy, such as immune checkpoint inhibitors, may be more effective for tumors with high immune cell infiltration (87). Other strategies, such as immune modulators or cell therapies, may be needed for tumors in immune deserts to attract more immune cells to the tumor microenvironment (88, 89).

However, this study also has limitations. First, this study’s analysis data come from public data resources such as TCGA, ICGC, and GEPIA (90). They lack their sequencing data. Specific clinical cases need to be collected for experiments to verify the credibility of the constructed prognostic model in predicting the prognosis of HCC patients. In addition, the data sources in public databases also have limitations. The experimental techniques used in different studies, such as different sequencing platforms or chip technologies, may be different. These technical differences will lead to incomparability between data. The data processing and standardization methods may vary, affecting the consistency of the data. Differences in the geographical origin, collection time, and sample processing methods will also introduce variability. Some gene expression data may have missing values, resulting in incomplete data. At the same time, biological differences between different samples in sequencing data, such as individual differences, mixed cell types, etc., may lead to variability in gene expression. Different conditions for sample collection and processing (such as temperature, culture medium, etc.) will affect gene expression. We use different databases and statistical schemes to verify each other to minimize the differences caused by different databases and sequencing batches. Secondly, this study lacks sufficient *in vitro* or *in vivo* experiments to explore the molecular mechanisms by which differentially expressed inflammation-related genes in liver cancer affect the prognosis of HCC patients to confirm the reliability of the GO and KEGG enrichment analysis results of differentially expressed inflammation-related genes in this study. Therefore, Many experiments are needed to prove the mechanical connection between the differential expression of inflammation-related genes and HCC proliferation and metastasis. Finally, the underlying specific mechanisms between differentially expressed inflammation-related genes in HCC and tumor immunity are poorly understood and require further experimental and clinical studies to verify.

## 5 Conclusion

The mRNA expression of six inflammation-related genes (C3, CTNNB1, CYBC1, DNASE1L3, IRAK1, and SERPINE1) is closely related to the overall survival rate, tumor immune infiltration, and clinical stage of HCC patients. Among them, CYBC1 is an independent risk factor affecting the prognosis of HCC patients. These findings provide a basis for the pathogenesis and clinical treatment of HCC and improve treatment strategies and early screening for HCC patients.

## Data availability statement

The original contributions presented in the study are included in the article/Supplementary material, further inquiries can be directed to the corresponding authors.

## Ethics statement

Ethical approval was not required for the studies on humans in accordance with the local legislation and institutional requirements because only commercially available established cell lines were used.

## Author contributions

YL: Writing – original draft. YF: Writing – original draft. DL: Writing – original draft. JW: Writing – review & editing. ZiH: Writing – review & editing. XueyL: Writing – review & editing. XuemL: Writing – review & editing. CW: Writing – original draft, Writing – review & editing. ZhH: Writing – original draft, Writing – review & editing.

## Footnotes

^1^ https://xenabrowser.net/datapages/

^2^ https://dcc.icgc.org/

^3^ https://bioinfogp.cnb.csic.es/tools/venny/
